# Identification of Growth Inhibitors of the Fish Pathogen *Saprolegnia parasitica* Using *in silico* Subtractive Proteomics, Computational Modeling, and Biochemical Validation

**DOI:** 10.3389/fmicb.2020.571093

**Published:** 2020-10-16

**Authors:** Sanjiv Kumar, Rahul Shubhra Mandal, Vincent Bulone, Vaibhav Srivastava

**Affiliations:** ^1^Division of Glycoscience, Department of Chemistry, School of Engineering Sciences in Chemistry, Biotechnology and Health, Royal Institute of Technology (KTH), AlbaNova University Centre, Stockholm, Sweden; ^2^Department of Cancer Biology, Abramson Family Cancer Research Institute, Perelman School of Medicine, University of Pennsylvania, Philadelphia, PA, United States; ^3^School of Agriculture, Food and Wine, The University of Adelaide, Adelaide, SA, Australia

**Keywords:** disease control, fish pathogen, growth inhibitors, oomycete, *Saprolegnia parasitica*, *in silico* proteomics

## Abstract

Many Stramenopile species belonging to oomycetes from the genus *Saprolegnia* infect fish, amphibians, and crustaceans in aquaculture farms and natural ecosystems. *Saprolegnia parasitica* is one of the most severe fish pathogens, responsible for high losses in the aquaculture industry worldwide. Most of the molecules reported to date for the control of *Saprolegnia* infections either are inefficient or have negative impacts on the health of the fish hosts or the environment resulting in substantial economic losses. Until now, the whole proteome of *S. parasitica* has not been explored for a systematic screening of novel inhibitors against the pathogen. The present study was designed to develop a consensus computational framework for the identification of potential target proteins and their inhibitors and subsequent experimental validation of selected compounds. Comparative analysis between the proteomes of *Saprolegnia*, humans and fish species identified proteins that are specific and essential for the survival of the pathogen. The DrugBank database was exploited to select food and drug administration (FDA)-approved inhibitors whose high binding affinity to their respective protein targets was confirmed by computational modeling. At least six of the identified compounds significantly inhibited the growth of *S. parasitica in vitro*. Triclosan was found to be most effective with a minimum inhibitory concentration (MIC_100_) of 4 μg/ml. Optical microscopy showed that the inhibitors affect the morphology of hyphal cells, with hyper-branching being commonly observed. The inhibitory effects of the compounds identified in this study on *Saprolegnia*’s mycelial growth indicate that they are potentially usable for disease control against this class of oomycete pathogens. Similar approaches can be easily adopted for the identification of potential inhibitors against other plant and animal pathogenic oomycete infections.

## Introduction

Most species from the oomycete genus *Saprolegnia* are opportunistic parasites (Plumb, 1999) that cause the disease saprolegniasis in various fish species (van West, 2006), amphibians (Blaustein et al., 1994), crustaceans (Diéguez-Uribeondo et al., 1994), and other aquatic animals (Fernandez-Beneitez et al., 2011). The virulent strains of *Saprolegnia parasitica* infect eggs, juvenile, and adult fish, leading to important losses in fish farms worldwide (van West, 2006). The chemical malachite green was used effectively for the treatment of saprolegniasis (Willoughby and Roberts, 1992) until it was banned in 2002 by the United States and several other countries due to its carcinogenic and mutagenic effects in humans and animals (Srivastava et al., 2004). The aquaculture production is increasing every year, and due to the lack of suitable and effective chemicals to control saprolegniasis, a re-emergence and spread of the disease is observed worldwide. Losses due to *S. parasitica* are higher in fish farms than in wild environments because the farmed animals are typically held in high densities and exposed to constant stress and various types of pollutants, factors that altogether increase the risk of infection and spread of the disease.

To date, multiple chemicals have been trialed to control saprolegniasis (Table 1), but their negative impact on the environment or the health of fish farmers or the fish hosts typically prevents their practical use in aquaculture. Two main classes of molecules have been investigated that have either general antimicrobial activity or a more targeted mode of action. From the first class, the least harmful compound is sodium chloride, but this salt is efficient only at high concentrations, which makes its use impractical in freshwater (Das et al., 2012). Hydrogen peroxide also has minimal impact on the environment and has been used to efficiently treat *Saprolegnia* infections in catfish (Howe et al., 1999). More recently, however, its application has been shown to provoke negative side effects, such as reduced fish growth and damage to the gills (Mustafa, 2019). Likewise, while boric acid inhibits germination and colonization of spores as well as mycelial growth in *Saprolegnia* species (Ali et al., 2014), an excess of boron affects DNA integrity of blood and sperm cells in Nile tilapia (Acar et al., 2018) and adversely impacts the growth of rainbow trout (Öz et al., 2018). Peracetic acid is another nonspecific antimicrobial chemical with proven activity against *S. parasitica* (Marchand et al., 2012), but its use in fish farms is hindered by the fact that it causes lacrimation at low concentrations as well as extreme discomfort and irritation of the upper respiratory tract in humans (N. R. Council, 2010). Other efficient compounds have adverse effects on the environment. Examples are formaldehyde, sometimes used for the treatment of eggs and larval stages (Gieseker et al., 2006), and copper sulfate, which inhibits *Saprolegnia* growth by affecting multiple biological functions, including protein and energy biogenesis (Hu et al., 2016).

**Table 1:** List of compounds reported earlier to be effective in controlling the growth and spread of *S. parasitica* in aquaculture.

Drugs	Effective concentration	Target/Mechanism	References
NaCl	~15 mg/ml	Unknown	Marking et al., 1994; Das et al., 2012
Formalin	0.1 mg/ml	Unknown	Gieseker et al., 2006; Khodabandeh and Abtahi, 2006
Boric acid	0.5 mg/ml	Germination and colonization	Ali et al., 2014
Peracetic acid	4–10 μg/ml	Unknown	Marchand et al., 2012
Hydrogen peroxide	50–250 μg/ml	Unknown	Mitchell and Collins, 1997
Clotrimazole	~1–2 μg/ml	CYP51 sterol 14α-demethylase	Warrilow et al., 2014
Copper sulfate	0.5 μg/ml	Protein and energy biogenesis	Sun et al., 2014
Bronopol	30 μg/ml	Dehydrogenase inhibitor	Pottinger and Day, 1999; Branson, 2002
Saprolmycins A–E angucycline antibiotic	3.9–7.8 ng/ml	Unknown, probably histidine kinases inhibitor	Nakagawa et al., 2012
Chitosans	~0.4 mg/ml	Fungicidal	Muzzarelli et al., 2001
Oridamycins	3.0 μg/ml	Unknown	Takada et al., 2010
Dioscin	2.0 μg/ml	Damages mycelium, accumulation of reactive oxygen species	Liu et al., 2015
Amphotericin B	5.0 μg/ml	Sterol biosynthesis	Bly et al., 1996
Cladomarine/Cladosporin	13–23 μg/ml	Probable lysyl-tRNA synthetase inhibitor	Takahashi et al., 2017
Malachite green	1.0 μg/ml	Affects multiple proteins	Martin, 1968; Willoughby and Roberts, 1992
Nikkomycin Z	25–99 μg/ml	Chitin synthases inhibitor	Guerriero et al., 2010
Benzoic acid	100 μg/ml	Unknown	Tedesco et al., 2019

Other chemicals with more specific modes of action have been tested against *Saprolegnia* species. For example, bronopol (2-bromo-2-nitropropane-1,3-diol) and its methyl derivative 2-methyl-4-isothiazolin-3-one oxidize thiol groups in proteins and are, as such, inhibitors of dehydrogenases. These chemicals have been identified as promising compounds for the control of *Saprolegnia* infections in ova and adult fish (Branson, 2002; Oono and Hatai, 2007), although tolerant strains of *Saprolegnia* have been reported (Rezinciuc et al., 2014). Further chemicals of the oxyalkylchalcone class have been developed to overcome bronopol resistance. Promising molecules with higher inhibitory activity than bronopol on *Saprolegnia* growth are 2',4'-dihydroxychalcone and its oxyalkylated derivative produced by chemical synthesis, 2-hydroxy, 4-farnesyloxychalcone (Flores et al., 2016). However, these molecules were tested *in vitro* only and there are no results that demonstrate both their efficiency in infected animals and innocuousness to the environment. An attractive alternative approach to the chemical synthesis of novel inhibitors is the search for natural molecules that exhibit antimicrobial properties. Relevant examples that have been shown to be effective against *S. parasitica* with weak or no activity against fungi, bacteria, microalgae, or zooplankton are the angucycline antibiotics saprolmycins A and E from *Streptomyces* sp. (Nakagawa et al., 2012). Likewise, cladomarine and quellenin, two antibiotics produced by the deep-sea fungus *Penicillium coralligerum* (YK-247) and *Aspergillus* sp. (strain YK-76), respectively, were shown to exhibit anti-*Saprolegnia* activity (Takahashi et al., 2017). However, despite the discovery of these promising natural anti-oomycete compounds, their large-scale production has proven to be challenging and too expensive to be commercially viable.

**Figure 1:**
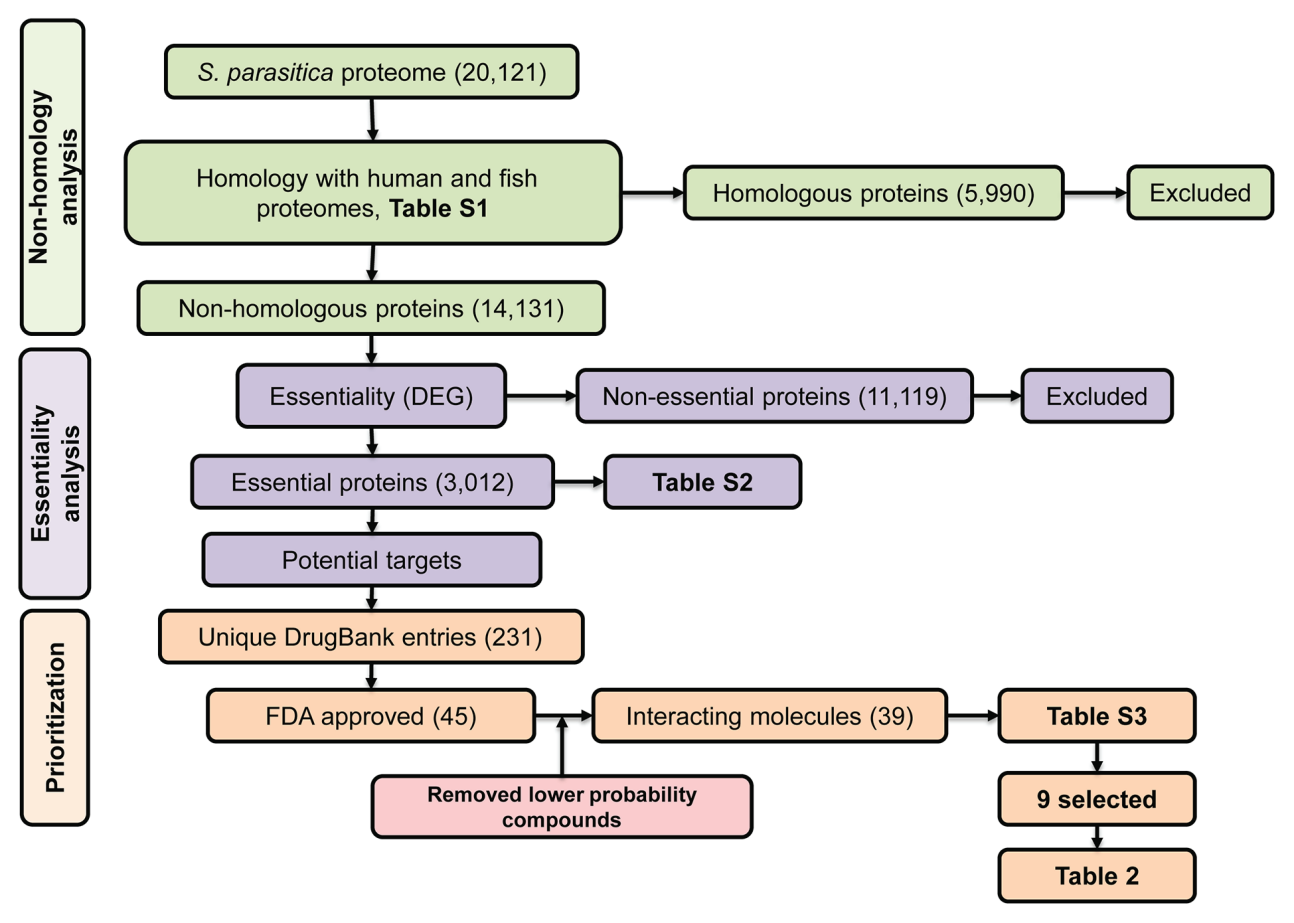
Schematic representation of the subtractive proteomics approach used in the present study. The whole proteome of *Saprolegnia parasitica* was searched using blastp (E-value < 1.0E-03, bitscore > 100) against human and fish proteomes followed by Database of Essential Genes (DEG) and finally, against DrugBank database for identification of food and drug administration (FDA)-approved inhibitor compounds.

Despite the numerous efforts mentioned above, we are still lacking compounds that can be used in the aquaculture industry to tackle saprolegniasis. Bioinformatic approaches have allowed the identification of inhibitors of bacterial pathogens of humans (Solanki and Tiwari, 2018; Bhardwaj et al., 2019; Cesur et al., 2020). Inspired by these successful strategies, here, we aim to identify specific protein targets and potential lead inhibitors of *S. parasitica* using combined *in silico* subtractive proteomics with a sequence-based approach. Our strategy presented in Figure 1 consisted in the analysis of the whole predicted proteome of *S. parasitica* and its comparison with fish and human proteomes to identify non-homologous essential proteins from the pathogen. The DrugBank database was subsequently searched to select potential food and drug administration (FDA)-approved inhibitors of these proteins, which were subsequently tested *in vitro* for their efficacy in arresting the growth of *S. parasitica*. With this approach, we have identified several inhibitors that exhibit MIC_100_ as low as 4 μg/ml. Their interactions with their protein targets were also studied by computational modeling, providing further evidence of the specific nature of the observed inhibitory effects. Similar strategy to identify and test novel compounds can be extended to other pathogenic oomycetes.

## Materials and Methods

### Identification of Non-homologous Essential Proteins

The predicted proteome of *S. parasitica* (strain CBS 223.65) was retrieved from the NCBI website^1^ using genome assembly ASM15154v2. Conserved protein domains were predicted using pfam v31.0 (Finn et al., 2016) and conserved domain database (CDD; Marchler-Bauer et al., 2017). Protein sequences deduced from the human (*Homo sapiens* GRCh38.p11) and 15 fish genomes (Supplementary Table S1) were compared with the sequences from *S. parasitica* using blastp (BLAST 2.2.31+). The *Saprolegnia* proteins that did not present any significant sequence similarity (E-value < 1.0E-03, bitscore > 100) with any of the human or fish proteins were considered as non-homologous and used for further analysis. Their sequences were searched against the Database of Essential Genes (DEG v15.2; Luo et al., 2014) using blastp to create a list of essential proteins from *S. parasitica*. The DEG database comprises genes from *archaea*, bacteria, and eukaryotes that are known to be vital for the organism considered. As it is generally assumed that proteins essential to one organism are also likely to be essential to other organisms, proteins from *S. parasitica* similar to proteins from the DEG database, i.e., proteins with an E-value < 1.0E-05 and a bitscore > 100, were considered as potential targets of anti-oomycete compounds and used for further analysis.

### Druggability Potential of Non-homologous Essential Proteins

An effective way to estimate the druggability of a protein, i.e., its capacity to bind one or several inhibitors, is to identify in the first instance similar proteins that have been shown to interact with these inhibitors (Keller et al., 2006). The DrugBank database (v5.1.1; Wishart et al., 2018) is a unique collection of bioinformatic and cheminformatic resources that combines drug data with information on the corresponding protein targets. The non-homologous essential proteins from *S. parasitica* identified as described above were searched against the DrugBank database using blastp. The proteins that showed significant scores (E-value < 1.0E-05, bitscore > 100) and their corresponding FDA-approved compounds were further considered.

### Homology Modeling and Docking

Homology modeling was performed using the CPHmodels 3.2 Server (Nielsen et al., 2010) that uses profile-profile alignment guided by protein secondary structure and exposure predictions for template identification. The generated models were further minimized using Steepest Descent and the conjugate gradient method in Discovery Studio 2.5 (Biovia, 2018). The models were used for molecular docking with selected lead compounds using AutoDock Vina (Trott and Olson, 2010) in PyRx (Dallakyan, 2008) with default parameters. The 3D structures of the active compounds were retrieved from the DrugBank database and minimized in PyRx before docking. The docked complexes were further visualized using PyMol (Seeliger and de Groot, 2010) and LigPlot+ (Laskowski and Swindells, 2011), and the corresponding binding energies were calculated using AutoDock Vina (Trott and Olson, 2010).

### *Saprolegnia* Cultures and Test of Growth Inhibitors

*S. parasitica* (strain CBS223.65) was maintained on potato dextrose agar (PDA) by inoculating Petri dishes with agar plugs carrying pre-grown mycelium. The cultures were kept at 24°C in the dark, and inoculations were repeated every 15 days. Growth inhibition experiments were conducted in the synthetic medium of Machlis (1953). First, sesame seeds carrying *S. parasitica* mycelium were prepared by spreading autoclave-sterilized seeds (121°C for 20 min) on PDA plates that contained a central agar plug covered with mycelium. After 5 days at 24°C, the seeds coated with hyphal cells were transferred to 24-well flat-bottom tissue culture plates (one seed per well) containing 1 ml Machlis medium in each well. Dilutions of the selected compounds were used to determine the effective concentration of each inhibitor (Table 2). Copper sulfate (Sun et al., 2014), boric acid (Ali et al., 2014), benzoic acid (Tedesco et al., 2019), and malachite green (Martin, 1968), all known to be effective inhibitors of *S. parasitica*, were used as positive controls. Solvents of each class of inhibitor, i.e., sterile water, dimethyl sulfoxide (DMSO), ethanol, and acetone, were used as negative controls. Varying concentrations of each solvent (0.01, 0.1, 1.0, and 10.0%) were also tested to determine whether they had any effect on the growth of the mycelium (Supplementary Figures S1A,B). All concentrations were tested in three or more replicates. The plates were incubated at 24°C for 4 days, and the extent of mycelial growth in each well was observed and measured daily (colony diameter). MIC_100_ were defined as the inhibitor concentrations for which no growth was observed in four independent replicates after 4 days of incubation at 24°C.

**Table 2:** *In-vitro* screening of the selected compounds on the growth of *S. parasitica* hyphal cells.

Compounds	Solvent	MIC range (μg/ml)	MIC in Machlis medium (μg/ml)	MIC in PDA plates (μg/ml)	Microscopy (μg/ml)	SPRG targets	References
Triclosan	Acetone	4–6	6	4	4	SPRG_20215	This study
Acetohydroxamic acid	Water	600–800	800	600	600	SPRG_06801	This study
Sulfamethoxazole	DMSO	800	800	800	400	SPRG_19504	This study
Chloramphenicol	Ethanol	800	800	800	800	SPRG_35021	This study
Azelaic acid	Water	800	800	800	800	SPRG_13682	This study
Benzoic acid	Water	400–600	600	400	400	SPRG_02663	This study, (Tedesco et al., 2019)
Glycerol	Water	>1,000	ND	ND	ND	SPRG_01876	This study
Albendazole	DMSO	>1,000	ND	ND	ND	SPRG_07119	This study
Thiabendazole	DMSO	>1,000	ND	ND	ND	SPRG_07119	This study
Copper sulfate	Water	400	400	400	200	Control	(Sun et al., 2014)
Boric acid	Water	400	400	400	400	Control	(Ali et al., 2014)
Malachite green	Water	0.8–1.0	1	0.8	0.8	Control	(Martin, 1968)

ND, not determined.

**Figure 2:**
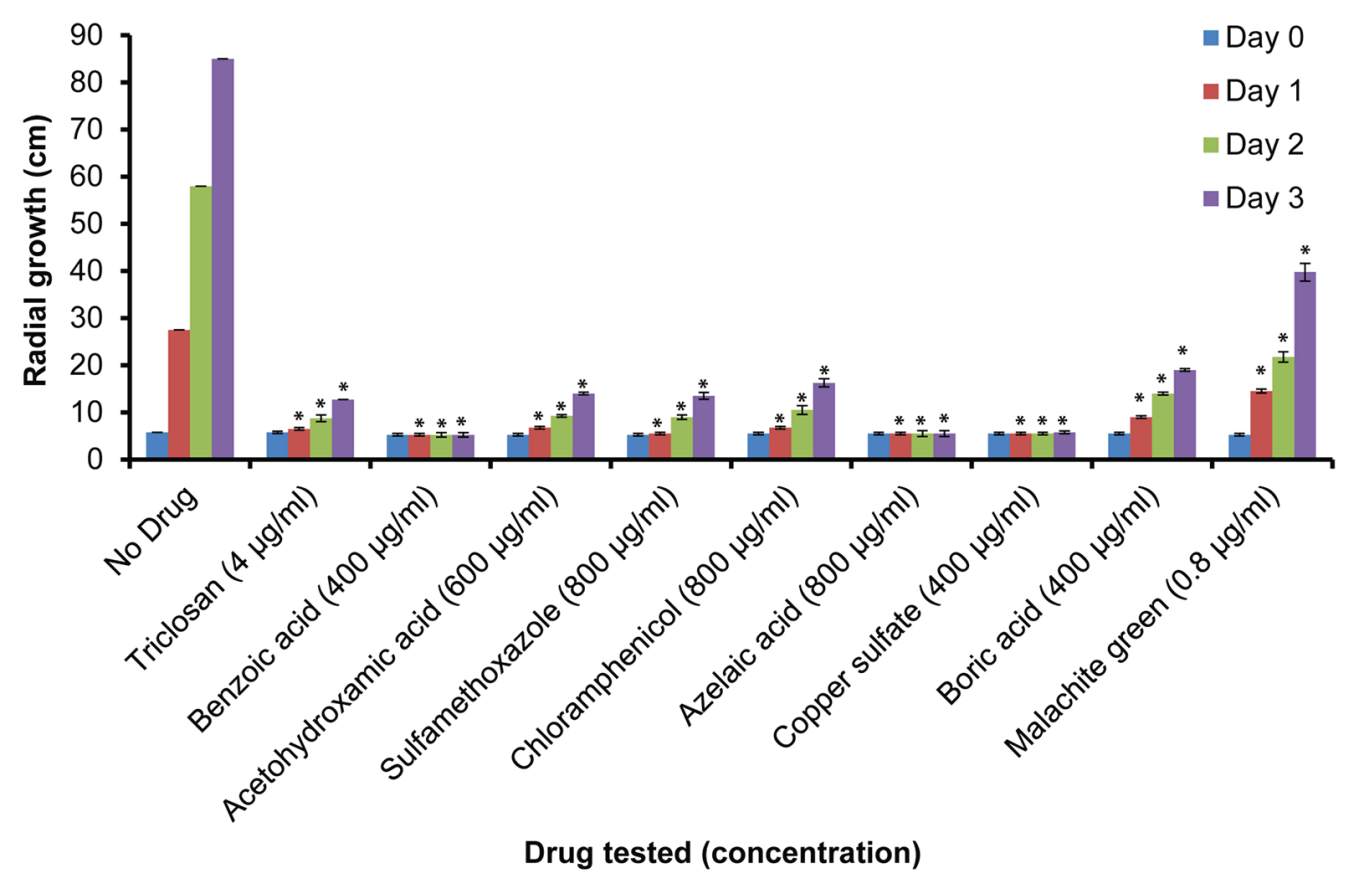
Relative growth of *S. parasitica* on potato dextrose agar (PDA) plates. Growth of mycelium (measured as diameter in cm) in the presence of sub-inhibitory concentrations of the tested compounds after 0 (day 0), 24 (day 1), 48 (day 2), and 72 h (day 3) of culture at 24°C. Four independent replicates were measured for each drug. A Student’s *t*-test was also performed to check significant differences between drug treated samples compared to the sample without drug (^*^p < 0.0001).

Additional growth inhibition experiments were conducted on PDA plates. These were prepared using the MIC_100_ determined in the Machlis medium and the same positive and negative controls as listed above (Supplementary Figure S2). Mycelial growth was quantified by measuring the diameter of the colonies on the PDA plates pre-inoculated with single agar plugs (5 mm) placed at the center of the plates. Experiments were conducted in triplicates and measurements of colony diameters were made at 0 (day 0), 24 (day 1), 48 (day 2), and 72 h (day 3) of culture at 24°C (Figure 2).

### Optical Microscopy

The effect of the tested compounds on the morphology of hyphal cells was observed by optical microscopy in 24-well flat-bottom tissue culture plates which contained 1 ml Machlis medium and appropriate dilutions of the lead compounds. Controls were performed in parallel as indicated above. Observations were made using a Leica Microsystems Ltd. DFC295 optical microscope, and images were recorded using the Leica Application Suite software v4.1.0.

## Results and Discussion

### Identification of Non-homologous Essential Proteins in *S. parasitica* and *in silico* Selection of Growth Inhibitors

To identify essential pathogen-specific proteins and potential specific growth inhibitors of *S. parasitica*, the sequences of all predicted proteins from the pathogen were compared with human and fish protein sequences (Supplementary Table S1). Among the 20,121 proteins encoded by the genome of *S. parasitica*, 5,990 were similar to human or fish proteins and were therefore disregarded for further analysis (Figure 1). Out of the remaining 14,131 non-homologous proteins, 3,012 showed significant similarity with essential proteins in the DEG database. These proteins were considered essential for *S. parasitica* and used for further analysis (Supplementary Table S2). Out of these, 117 were similar to target sequences in the DrugBank database, yielding 231 DrugBank unique entries, of which 45 are FDA approved (Supplementary Table S3). As the DrugBank database enlists not only inhibitors for their respective proteins but also interacting compounds, six of these 45 entries (DB01987, DB03147, DB03247, DB00140, DB09510, and DB09513) were unlikely to be efficient inhibitors. Hence, the list of potential inhibitors of growth was narrowed down to 39 compounds (Supplementary Table S3). These were further subdivided into two groups: the first group gathers compounds that potentially interact with two or more proteins from *S. parasitica*, whereas the second group consists of molecules that are predicted to bind one protein only (Supplementary Table S3).

### Compounds Interacting With Two or More Essential Proteins From *S. parasitica* (Group 1)

This group contains eight compounds that can potentially interact with 29 proteins from *Saprolegnia* (Supplementary Table S3). Interestingly, among these molecules, three are semisynthetic echinocandin derivatives, namely caspofungin, anidulafungin, and micafungin. Echinocandins are well-characterized antifungal agents (Gil-Alonso et al., 2015; Perfect, 2017) that inhibit 1,3-β-d-Glucan synthases, i.e., the enzymes responsible for the formation of the major cell wall β-glucans in fungi. Earlier work from our group has shown that 1,3-β-d-glucans are major components of the cell wall of *S. parasitica* and other oomycete species (Bulone et al., 1990; Bulone and Fèvre, 1996; Mélida et al., 2013). 1,3-β-Glucan synthases have no mammalian homologues and are therefore ideal targets for antifungal and anti-oomycete inhibitors (Perfect, 2017). CDD analysis of the *S. parasitica* proteome led to the identification of at least 10 proteins that belong to the glucan synthase family, including SPRG_05460, which was not identified in our *in silico* screening as an echinocandin-binding protein. We did test multiple echinocandins in previous work and showed that they do not inhibit the 1,3-β-Glucan synthases of *Saprolegnia in vitro* and are therefore unlikely to stop the growth of this class of micro-organisms (Bulone, 1992). This probably reflects different properties between the fungal and oomycete enzymes as one would expect given their low level of similarity, which our blast results revealed to be of 29–34%.

Picric acid, citric acid, and artenimol are three other compounds from this group of inhibitors. However, their specificity is predicted to be low, essentially because of their general antimicrobial activity. Indeed, picric acid has been used as an antiseptic for a long time (Tidy, 1915) and is predicted to inhibit at least eight proteins from *S. parasitica* (Supplementary Table S3). The same applies to citric acid, which we have predicted to interact with at least four proteins from *S. parasitica*, including two uncharacterized proteins (SPRG_06530, a probable FtsZ protein, and SPRG_11850 and a phosphoribosylaminoimidazole carboxylase), a tryptophan synthase (SPRG_05079) and a cell division protein FtsZ (SPRG_11942). Tryptophan synthase catalyzes the final step of the tryptophan biosynthetic pathway. This enzyme is absent in mammals, which makes it an attractive target for many other pathogens (Shen et al., 2009). The cell division protein FtsZ has been described as a potential drug target in various bacterial pathogens (Shen et al., 2009), and phosphoribosylaminoimidazole carboxylase has been proposed previously as a target for drug therapy against *Candida albicans* (Donovan et al., 2001). Despite these observations, the diversity of enzymes and non-catalytic proteins to which citric acid potentially binds to suggests that its mode of action is not specific. Similarly, artenimol (dihydroartemisinin) is predicted to interact with at least two different *Saprolegnia* proteins, namely SPRG_00366, a predicted pyridoxal biosynthesis lyase (PdxS), and SPRG_09913, a negative regulator of GroEL domains. Interestingly, this compound has been shown to be active against a range of organisms as diverse as viruses (Efferth et al., 2008), malarial protozoa (Pull et al., 2019), plant pathogenic fungi (Tang et al., 2000), and even certain tumor cells (Jansen et al., 2011) but its efficacy against oomycetes has not been reported.

Another compound identified in group one is formic acid. It is predicted to interact with at least three chorismate binding enzymes, an anthranilate synthase protein (SPRG_06062), and two hypothetical proteins (SPRG_12737 and SPRG_16397). The antimicrobial activity of formic acid has been reported on various bacterial and yeast pathogens (Plumed-Ferrer and von Wright, 2011; Lastauskiene et al., 2014). However, chronic exposure to this molecule has been shown to have adverse health effects similar to those observed in methanol poisoning, i.e., metabolic acidosis and ocular toxicity (Thompson, 1992).

α-Phosphoribosylpyrophosphoric acid is predicted to interact with two proteins of unknown functions and anthranilate phosphoribosyltransferase, which is involved in the biosynthesis of aromatic amino acids. This compound is a key intermediate of the biosynthesis of nucleotides and likely to interfere with other vital processes of the fish hosts. This suggests that it would not be suitable for the management of saprolegniasis.

In conclusion, the compounds from this first group of inhibitors are expected to lack specificity (e.g., citric acid) or to be inactive against *Saprolegnia* (e.g., echinocandins), and, in some cases, to have adverse health effects on the fish hosts or humans (e.g., formic acid). Thus, in order to select more specific inhibitors potentially usable against *Saprolegnia*, we have focused the rest of our study on molecules that are predicted to bind a single essential protein in the pathogen.

### Compounds Interacting With a Single Protein From *S. parasitica* (Group 2)

This group comprises 31 compounds, the majority being sulfonamides or “sulfa drugs” (18 molecules). Sulfonamides are predicted to interact with the protein SPRG_19504 in *S. parasitica*, which belongs to the dihydropteroate synthase (DHPS) family and contains a 7,8-dihydro-6-hydroxymethylpterin-pyrophosphokinase (HPPK) domain and a pterin-binding domain. In several bacteria and lower eukaryotes, HPPK (EC 2.7.6.3) and DHPS (EC 2.5.1.15) enzymes catalyze sequential reactions in the folic acid biosynthetic pathway (Illarionova et al., 2002). Higher eukaryotes obtain folate from dietary sources and lack necessary enzymes for folate biosynthesis (Matherly, 2001), whereas eubacteria and lower eukaryotes are able to synthesize tetrahydrofolate. Thus, the folate pathway is an interesting potential target for anti-infective agents. Indeed, DHPS is a well-known target of the sulfonamide class of antibacterial drugs (Hevener et al., 2010). CDD analysis of the proteome of *S. parasitica* revealed the presence of two additional proteins involved in the last step of the folic acid biosynthetic pathway and annotated as dihydrofolate reductase (DHFR), i.e., SPRG_10565 and SPRG_17339. These observations suggest that sulfonamides are potential effective inhibitors of the growth of *Saprolegnia*.

Quaternary ammonium compounds like cetrimonium and didecyldimethylammonium are predicted to interact with SPRG_00820, a hypothetical protein that contains a mycolic acid cyclopropane synthetase domain. An antimycotic activity was reported for cetrimide, in particular, against fungal keratitis caused by *Fusarium solani*, without any negative effect on eye corneal tissues (Mahmoud, 2007). Similarly, didecyldimethylammonium chloride (DDAC) is able to inactivate photosystem II (PSII) and disintegrate marine phytoplankton species (van Slooten et al., 2015). It was also found effective against *Escherichia coli* where it induced the formation of blebs in the cell membrane, causing leakage of the intracellular compartment and subsequent cell death (Yoshimatsu and Hiyama, 2007).

Triclosan and triclocarban are commonly used broad-spectrum antibacterial/antifungal agents that block fatty acid biosynthesis by inhibiting enoyl-ACP reductase (Dhillon et al., 2015). Both molecules are predicted to interact with the protein SPRG_20215 in *S. parasitica*, an uncharacterized protein that is predicted to contain a short-chain dehydrogenase/reductase (SDR) domain. Triclosan has been found to be effective against various Gram-negative and Gram-positive bacteria, as well as *Mycobacteria* (Schweizer, 2001) and *Plasmodium falciparum* (Surolia and Surolia, 2001), where it targets dihydrofolate reductase (Bilsland et al., 2018). This inhibitor is also active against the parasite *Leishmania panamensis* with no toxicity toward mammalian cells at concentrations as high as 200 μg/ml (Otero et al., 2014).

The benzimidazoles albendazole and thiabendazole are predicted to interact with the protein SPRG_07119 which carries a FAD-binding domain and is structurally similar to fumarate reductase 2 (PDB 5GLG) from *Saccharomyces cerevisiae*. Benzimidazoles are commonly used as fungicides and anthelminthic drugs where they specifically inhibit microtubule assembly (Davidse, 1986).

Two other approved compounds from group 2 are acetohydroxamic acid (Ohta et al., 2001) and ecabet (Shibata et al., 1997), which inhibit ureases. In *Saprolegnia*, both drugs are predicted to interact with the protein SPRG_06801, which is predicted to be a nickel-dependent amidohydrolase that catalyzes the decomposition of urea into carbamate and ammonia. As such, this catabolic reaction represents an important source of nitrogen in bacteria, fungi, and plants. Urease activity has been reported in *Cryptococcus gattii* (Feder et al., 2015) and the recently discovered novel systemic fungal pathogen *Emergomyces africanus* (Lerm et al., 2017). Urease is considered an essential enzyme and is explored as a target for the development of novel antibacterial agents (Follmer, 2010). Our *in silico* proteome analysis suggests that *S. parasitica* contains urease and therefore that inhibitors of this enzyme are worth testing against the pathogen.

Azelaic acid is predicted to interact with SPRG_13682, a hypothetical protein with 5'-3' exonuclease activity, and an *N*-terminal resolvase-like domain. Azelaic acid has been found effective against various Gram-positive and Gram-negative bacterial pathogens by inhibiting several oxidoreductases and interfering with glycolysis (Nazzaro-Porro, 1987).

Chloramphenicol was also identified in group 2. It is a common antibacterial agent that acts by inhibiting protein synthesis (Xaplanteri et al., 2003) and is considered fungistatic (Rooke and Shattock, 1983). It has been reported to exert variable inhibitory activity on the growth of *Phytophthora* species (Panek and Tomsovsky, 2017). Indeed, resistant strains of *Phytophthora* have been reported (Ann and Ko, 1992) alongside sensitive strains.

The last three compounds in group 2 are tromethamine, glycerol, and benzoic acid (Supplementary Table S3). Tromethamine is predicted to interact with the hypothetical protein SPRG_04175, which contains a biotin and thiamin synthesis associated domain. Glycerol is predicted to interact with a histidinol dehydrogenase (SPRG_01876) and it has been explored previously for its antibacterial activity (Saegeman et al., 2008). Benzoic acid is a common food preservative used to prevent the growth of yeast and molds (Brown and Swinburne, 1971). It most likely acts by interacting with the putative hydrolase SPRG_02663, a hypothetical Xaa-Pro dipeptidyl-peptidase with a *C*-terminal non-catalytic domain. This compound showed activity against various stages of fungal development (Amborabé et al., 2002) and it has recently been reported to be effective against *S. parasitica* (Tedesco et al., 2019), but its mechanism of inhibition was not investigated in these studies.

In conclusion, most of the inhibitors from group 2 have been shown to be effective against other microbial pathogens, which support the validity of our approach, but a large majority of these compounds have never been tested against *Saprolegnia*. Therefore, the next step of our work involved the use of *in vitro* assays in the presence of representative chemicals of the different classes from group 2 to determine whether these selected inhibitors affect the growth of *S. parasitica* and to which extent.

### Effect of Selected Compounds on the Growth of *S. parasitica* in Liquid Medium

From the above list of DrugBank compounds predicted to interact with single proteins of *S. parasitica* (group 2), nine representative compounds were tested for their growth inhibitory effect on *Saprolegnia* mycelium (Table 2; Supplementary Figure S1). In addition, copper sulfate, boric acid, and malachite green were used as positive controls, and the solvents of the different inhibitors were tested as negative controls (see Materials and Methods section). The solvents used for the preparation of stock solutions of the selected compounds, i.e., water, dimethyl sulfoxide (DMSO), ethanol, and acetone, did not inhibit the growth of *S. parasitica* at concentrations up to 1% v/v (Supplementary Figures S1A,B). These results are in keeping with previous reports (Xue-Gang et al., 2013; Hoskonen et al., 2015). However, some growth inhibition was observed with DMSO and ethanol at concentrations higher than 2% v/v, while growth was not perturbed in acetone at concentrations as high as 10% v/v.

Triclosan and triclocarban are predicted to target the protein SPRG_20215. Triclosan was found to be very effective when tested in liquid Machlis medium, with an MIC_100_ concentration of 4 μg/ml (Supplementary Figure S1C), and significant levels of inhibition still observed at a lower concentration of 2 μg/ml. Benzoic acid (targeting the hypothetical protein SPRG_02663) and acetohydroxamic acid (targeting the putative urease SPRG_06801) arrested growth completely at 600 and 800 μg/ml, respectively. In addition, significant growth inhibition was still observed at lower concentrations (Supplementary Figures S1D,E). Recently, another group also reported benzoic acid as an effective compound against *S. parasitica* (Tedesco et al., 2019). However, in our experimental conditions, the 100 μg/ml concentration tested by Tedesco et al. (2019) had no visible effect on *Saprolegnia* growth (Supplementary Figure S1D). Other compounds such as the sulfonamide sulfamethoxazole (800 μg/ml), chloramphenicol (800 μg/ml), and azelaic acid (800 μg/ml) showed growth inhibition at less than 1,000 μg/ml (Supplementary Figures S1F,G,H). Furthermore, glycerol, albendazole, and thiabendazole did not show any significant inhibitory effect up to a concentration of 1,000 μg/ml (Supplementary Figures S1I,J,K). The compounds that significantly inhibited the growth of *S. parasitica* at concentrations lower than 1,000 μg/ml were considered effective, and their activity was further analyzed.

As expected and reported earlier, the positive controls, such as copper sulfate (400 μg/ml; Sun et al., 2014), boric acid (400 μg/ml; Ali et al., 2014), and malachite green (2 μg/ml; Martin, 1968; Supplementary Figures S1L,M,N) arrested the growth of mycelium in the liquid medium of Machlis. However, the use of sesame seeds to inoculate the culture medium leads to variable amounts and quality of mycelium per cell culture well (Stueland et al., 2005). This variability of inoculum size and quality is most likely responsible for the discrepancies reported in the literature with regard to the most efficient inhibitory concentrations of the different drugs.

### Effect of the Selected Compounds on Radial Growth of *S. parasitica* on Solid Medium

To further evaluate the effect of the selected drugs on the growth of *S. parasitica* on a solid medium, we prepared PDA plates supplemented with the minimum inhibitory concentrations of each drug determined in the *in vitro* assays in liquid Machlis medium (Table 2). As no growth was observed for any of the compounds tested, a lower (sub-inhibitory) concentration was used to test the effect of each inhibitor (Table 2; Figure 2; Supplementary Figure S2). As expected, normal growth was observed on PDA plates devoid of inhibitor. Similar to the observations in liquid medium (Supplementary Figure S1), no inhibition was observed in the presence of glycerol, albendazole, and thiabendazole (1,000 μg/ml each) after 72 h culture at 24°C (Supplementary Figures S2K,L,M). Significant levels of inhibition were observed in the presence of triclosan (4 μg/ml), benzoic acid (400 μg/ml), acetohydroxamic acid (600 μg/ml), sulfamethoxazole (800 μg/ml), azelaic acid (1,000 μg/ml), chloramphenicol (800 μg/ml), copper sulfate (400 μg/ml), boric acid (400 μg/ml), and malachite green (0.8 μg/ml; Figure 2; Supplementary Figure S2).

### Effect of the Selected Compounds on the Morphology of *S. parasitica*

**Figure 3:**
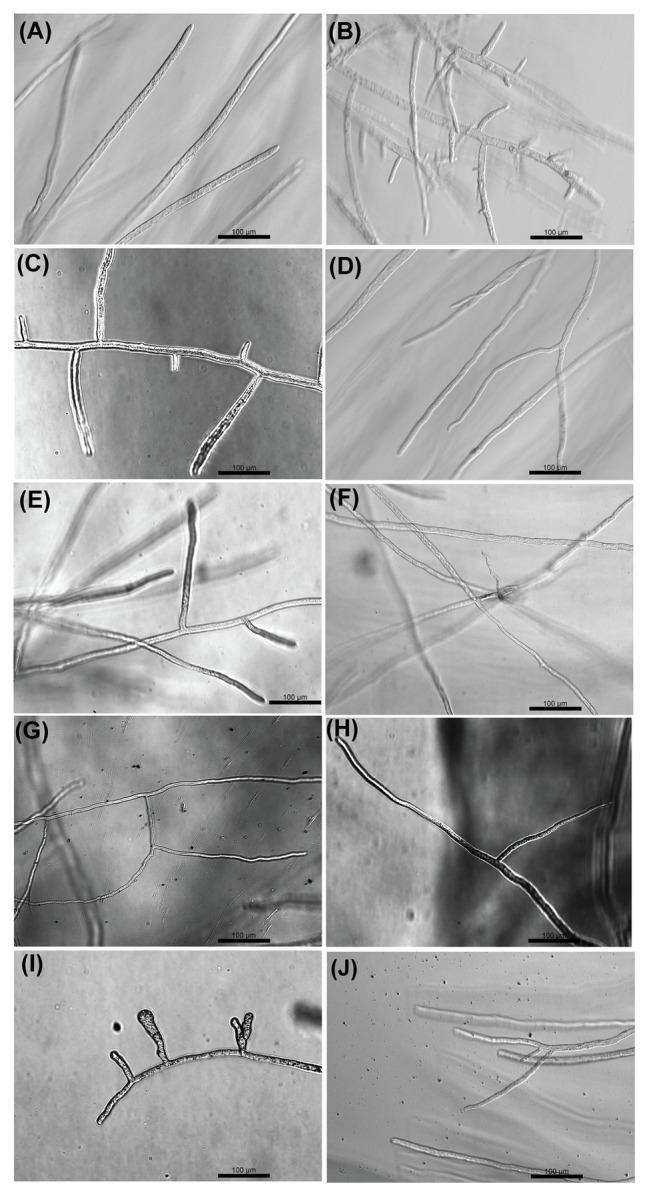
Optical microscopy observation of the effect of selected compounds on the growth of hyphae of *S. parasitica* after 24 h culture at 24°C in Machlis medium. **(A)** No Drug, **(B)** Triclosan (4 μg/ml), **(C)** Benzoic acid (400 μg/ml), **(D)** Acetohydroxamic acid (600 μg/ml), **(E)** Sulfamethoxazole (400 μg/ml), **(F)** Chloramphenicol (800 μg/ml), **(G)** Azelaic acid (800 μg/ml), **(H)** Copper sulfate (200 μg/ml), **(I)** Boric acid (400 μg/ml), and **(J)** Malachite green (0.8 μg/ml). Scale bars = 100 μm.

Optical microscopy observations were made to analyze the effect of the selected inhibitors on the morphology of *S. parasitica* (Figure 3). In the absence of inhibitor, the hyphae appeared as translucent elongated cells. As opposed to the control, hyper-branching was commonly observed in the presence of some of the drugs that arrest the growth of the mycelium (Figure 3). The level of hyper-branching varied for the different compounds and was dose-dependent (Supplementary Figure S3). Pronounced hyper-branching was observed in the presence of triclosan (4 μg/ml), followed by benzoic acid (400 μg/ml), sulfamethoxazole (400 μg/ml), and boric acid (400 μg/ml). In the presence of acetohydroxamic acid, chloramphenicol, azelaic acid, and copper sulfate, low levels of side branching were visible (Figure 3). Interestingly, compared to other strong inhibitors of growth, no pronounced hyper-branching was visible in the presence of malachite green (Figure 3J). Hyper-branching of the mycelium of *S. parasitica* in the presence of various inhibitors has not been explored previously. Therefore, an in-depth study of the mechanisms underlying the formation of such branching structures would be necessary to understand the effect of the inhibitors on cell development.

### Homology Modeling and Docking

**Figure 4:**
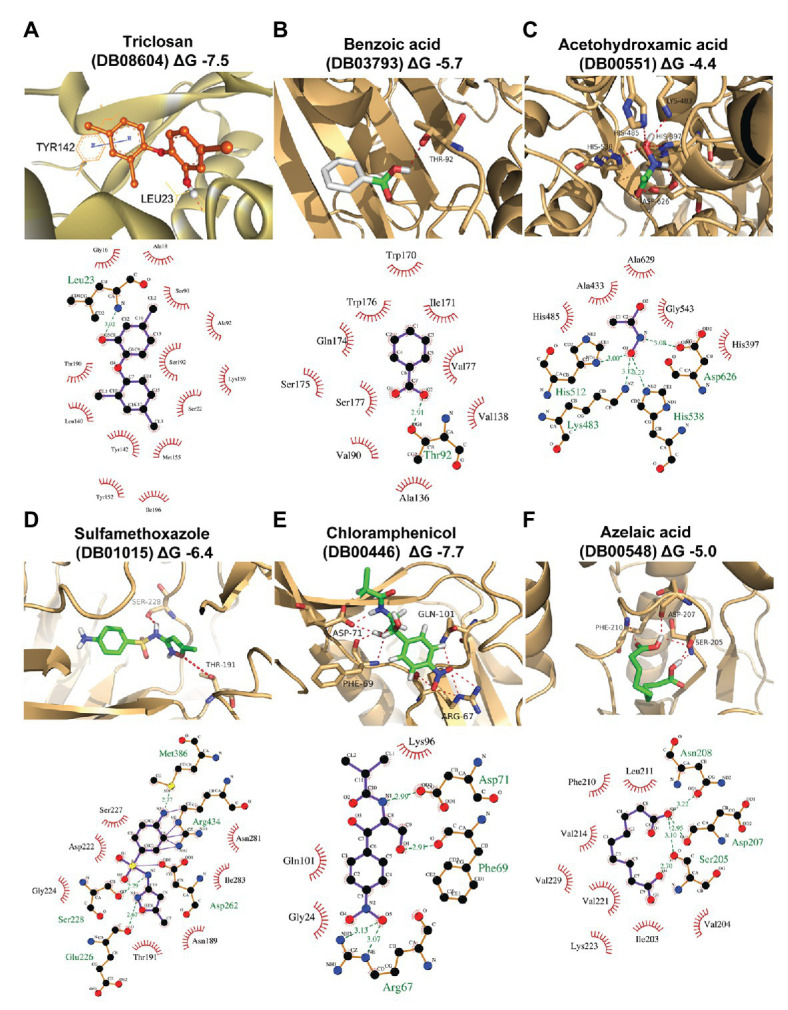
Homology modeling and docking of the tested compounds with their respective target proteins. For each of the protein-ligand complexes, the LigPlot cartoons show the interactions between each inhibitor and the amino acid residues in the active site cleft of the respective proteins. **(A)** SPRG_20215 with triclosan (DB08604; binding energy −7.5 kcal/mol), **(B)** SPRG_02663 with benzoic acid (DB03793; binding energy −5.7 kcal/mol), **(C)** SPRG_06801 (putative urease) with acetohydroxamic acid (DB00551; binding energy −4.4 kcal/mol), **(D)** SPRG_19504 with sulfamethoxazole (DB01015; binding energy −4 kcal/mol), **(E)** SPRG_35021 with chloramphenicol (DB00446; binding energy −7.7 kcal/mol), and **(F)** SPRG_13682 with azelaic acid (DB00548; binding energy −5.0 kcal/mol).

In this work, homology modeling and *in silico* docking studies were performed to better understand the mode of interaction between proteins and inhibitors. To this end, we used the six compounds shown to be effective in the *in vitro* assays along with their respective predicted target proteins (Figure 4; Supplementary Table S4). The six target proteins were SPRG_20215, SPRG_02663, SPRG_06801, SPRG_19504, SPRG_35021, and SPRG_13682. The 3D template structure for each of the selected proteins and corresponding percent sequence identity are presented in Supplementary Table S4. All proteins had a significantly high sequence identity of >30%, which allowed the generation of a reliable 3D protein structure, except for SPRG_02663, which has a slightly lower sequence identity (25.5%) with its template protein. The docking results show an approximate binding pattern of the inhibitors with their target proteins. Analysis of the docked complexes (compounds with their respective proteins) showed a considerable number of hydrogen bonding within the protein cavity or the active sites, which supports the specificity of the binding of each inhibitor with its respective protein. Furthermore, all the compounds showed significantly high binding energy toward their target, ranging from −7.7 to −4.4 kcal mol^−1^, which indicates strong interactions between the ligands and their targets (Figure 4; Supplementary Table S4).

The results of the virtual screening were visualized using LigPlot to show the details of the interactions of the proteins with their respective ligands (Figure 4). Triclosan (DB08604), which is predicted to bind to the uncharacterized protein SPRG_20215, interacts through a single H bond between the O5 atom of the drug and the backbone amino group of Leu23. In addition, there are 13 residues from the protein that contribute to hydrophobic interactions. Similar interacting residues have been reported earlier for an enoyl-acyl carrier protein reductase from *Thermus thermophilus* (Otero et al., 2012). The binding of benzoic acid (DB03793) to SPRG_02663 involves essentially hydrophobic interactions and a single H bond only. The SPRG_19504 protein complex with sulfamethoxazole (DB01015) involves seven amino acid residues from the protein that interact with the inhibitor through hydrophobic bonds while four residues are involved in H bonds. The interaction between SPRG_13682 and azelaic acid (DB00548) shows that the inhibitor interacts within the core hydrophobic pocket of the protein, with six hydrophobic residues involved in addition to the four H bonds associated with two polar and one negatively charged residues of the protein. Chloramphenicol (DB00446) interacts with SPRG_35021 through four H bonds while three other residues from the protein are engaged in hydrophobic interactions. The smallest inhibitor analyzed, acetohydroxamic acid (DB00551), forms H bonds with charged residues like His, Asp, and Lys, and it also interacts through hydrophobic bonds with five surrounding residues of the putative urease protein SPRG_06801. In summary, the docking study reveals that all complexes analyzed involve H bonding and hydrophobic interactions; it supports the initial screening results and provides initial evidence for the likely mechanisms of action of the six drug-proteins pairs identified in this work.

We have identified several compounds along with their probable target proteins using a subtractive proteomics approach and tested nine of these molecules *in vitro* for their efficacy against the growth of *S. parasitica’s* mycelium. Molecular docking studies showed that these compounds exhibit minimum binding energies to their respective targets and present a strong affinity toward the active pockets of the proteins, which further supports the experimentally observed growth inhibition. Considering both the low effective concentration of some of these compounds against *Saprolegnia* and the fact that they are already used in humans suggest that they would be safe to use in aquaculture. Moreover, the concentration of triclosan effective against *Saprolegnia* under our *in vitro* conditions (4 μg/ml) is significantly lower to its “no-observed-adverse-effect level” (NOAEL) in humans (0.3% w/w; Lee et al., 2019). Similarly, acetohydroxamic acid, which is effective against *S. parasitica* at around 600–800 μg/ml, is used at up to 15 mg/kg/day for the treatment of human urinary infections and stones (Griffith et al., 1981; Williams et al., 1984). Azelaic acid (600–800 μg/ml against *S. parasitica*) has not shown any toxic effect during oral (≤20 g/day) or topical (20% cream) administration to humans (Fitton and Goa, 1991). Both sulfamethoxazole and chloramphenicol have been widely used as antibacterial agents in humans and animals. Sulfamethoxazole (800 μg/ml against *S. parasitica*) has been used in combination with trimethoprim for over 30 years, with a recommended dosage of up to 40 mg/kg/12 h (Dudley et al., 1984). Similarly, also effective at 800 μg/ml against *S. parasitica*, chloramphenicol has a recommended dosage of 25 mg/kg/6 h (Powell and Nahata, 1982). Future work is required to confirm the probable mechanism of action of these promising compounds as well as their efficacy on the different developmental stages of *S. parasitica* (Srivastava et al., 2018). It may also become possible to increase their efficacy by downstream modification and make them usable for the treatment of saprolegniasis in fish eggs, juvenile, and adult fish. Furthermore, the current integrative approach is not limited to saprolegniasis but it can also be employed for the identification of potential inhibitors of other oomycete pathogens.

## Data Availability Statement

The datasets presented in this study can be found in online repositories. The names of the repository/repositories and accession number(s) can be found in the article/Supplementary Material.

## Author Contributions

SK, VB, and VS designed the experimental work, analyzed the data and wrote the manuscript. RM contributed to the molecular modeling experiments. All authors contributed to the article and approved the submitted version.

### Conflict of Interest

The authors declare that the research was conducted in the absence of any commercial or financial relationships that could be construed as a potential conflict of interest.

## Footnotes

^1^www.ncbi.nlm.nih.gov/genome

## Abbreviations

CDD: Conserved domain database
DDAC: Didecyldimethylammonium chloride
DEG: Database of essential genes
DHFR: Dihydrofolate reductase
DHPS: Dihydropteroate synthase
DMSO: Dimethyl sulfoxide
FDA: Food and drug administration
PDA: Potato dextrose agar
SDR: Short-chain dehydrogenases/reductases
