# Supplementary material for: Identification of Growth Inhibitors of the Fish Pathogen Saprolegnia parasitica Using in silico Subtractive Proteomics, Computational Modeling, and Biochemical Validation
Source: Front Microbiol. 2020 Oct 16;11:571093. doi: 10.3389/fmicb.2020.571093 (PMC7596660; doi:10.3389/fmicb.2020.571093)
Supplement: Supplementary file 1 [file Data_Sheet_1.pdf]

Supplementary Figures

for

Identification of growth inhibitors of the fish pathogen *Saprolegnia parasitica* using *in silico* subtractive proteomics, computational modelling and biochemical validation

Sanjiv Kumar, Rahul Shubhra Mandal, Vincent Bulone and Vaibhav Srivastava*

Effects of selected compounds on the growth of *S. parasitica*'s mycelium

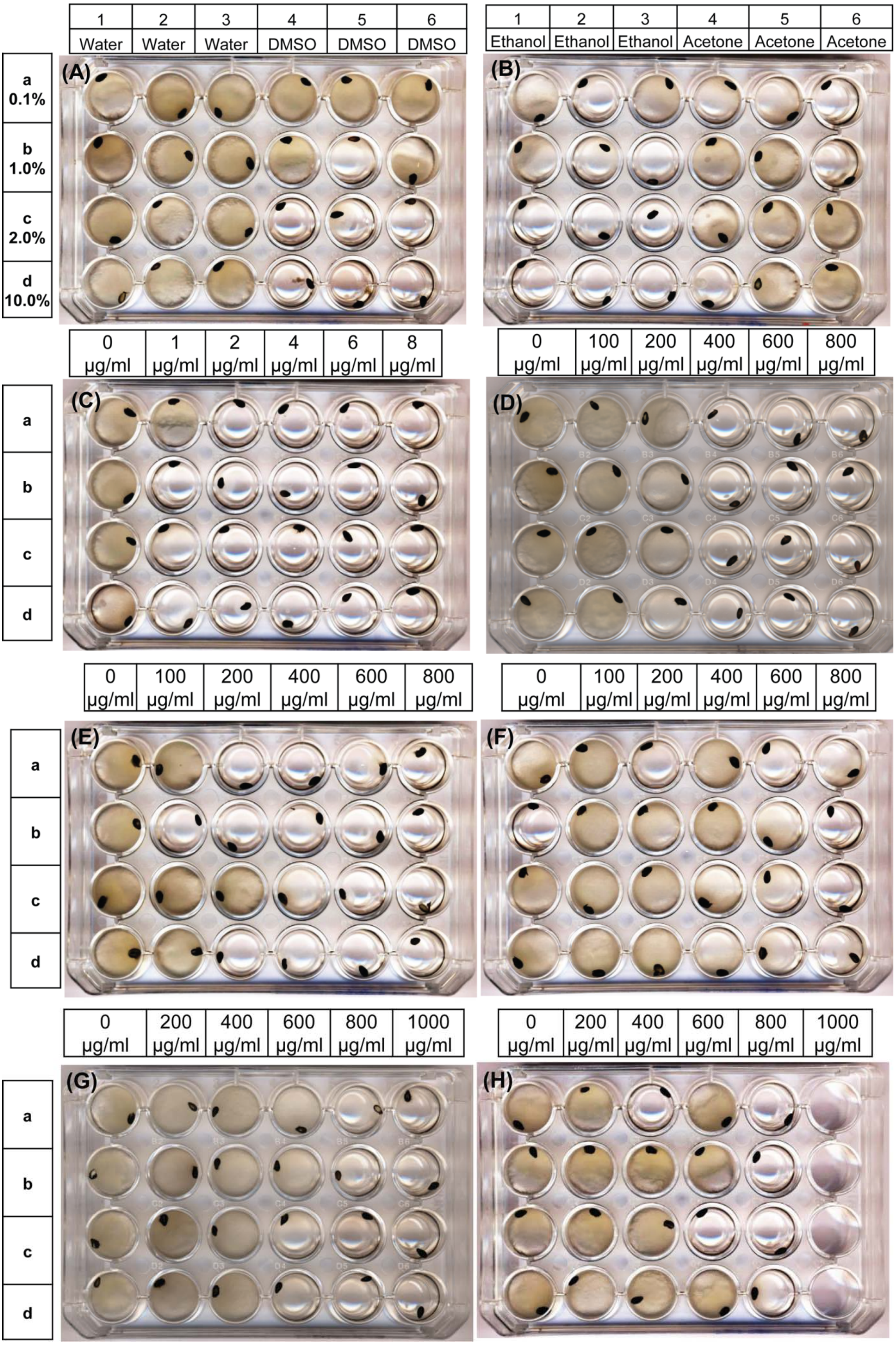

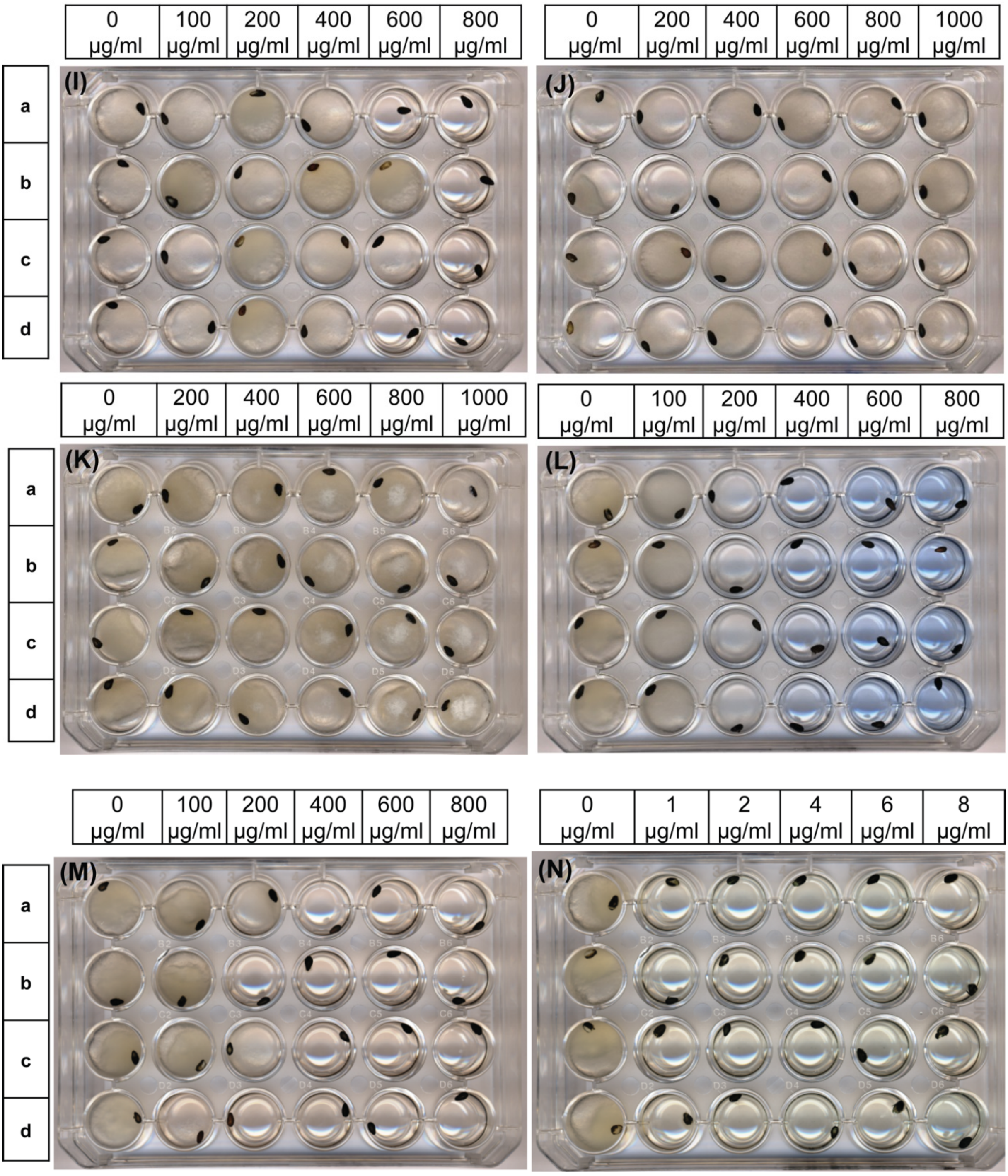

Supplementary Figure S1: Effects of selected compounds on the growth of *S. parasitica* mycelium in liquid Machlis medium. A: Water and DMSO control, B: Ethanol and acetone controls, C: Triclosan, D: Benzoic Acid, E: Acetohydroxamic Acid, F: Sulfamethoxazole, G: Chloramphenicol, H: Azelaic acid, I: Glycerol, J: Albendazole, K: Thiabendazole, L: Copper sulfate, M: Boric acid, N: Malachite green. Rows A-D in each panel represent 4 replicates of the conditions mentioned at the top of each plate.

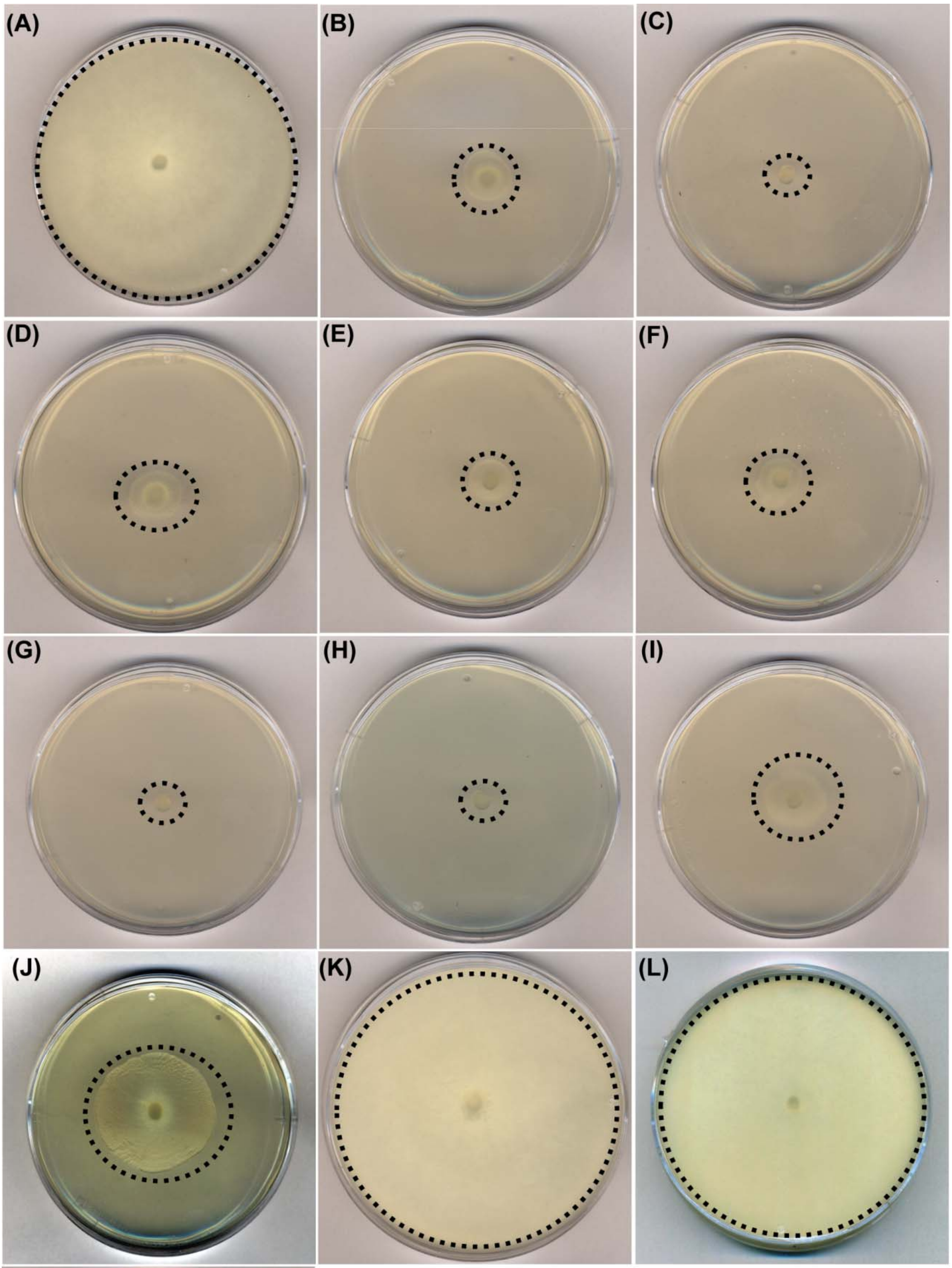

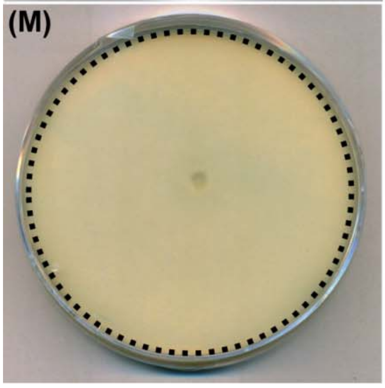

Supplementary Figure S2: Representative images showing the growth of *S. parasitica*'s mycelium on PDA plates in the presence of selected compounds after 48 h culture at 24°C. A: No Drug, B: Triclosan (4 µg/ml), C: Benzoic acid (400 µg/ml), D: Acetohydroxamic acid (600 µg/ml), E: Sulfamethoxazole (800 µg/ml), F: Chloramphenicol (800 µg/ml), G: Azelaic acid (800 µg/ml), H: Copper sulfate (400 µg/ml), I: Boric acid (400 µg/ml), J: Malachite green (0.8 µg/ml), K: Glycerol (1000 µg/ml), L: Albendazole (1000 µg/ml), M: Thiabendazole (1000 µg/ml). The growth area is marked by black dotted circle.

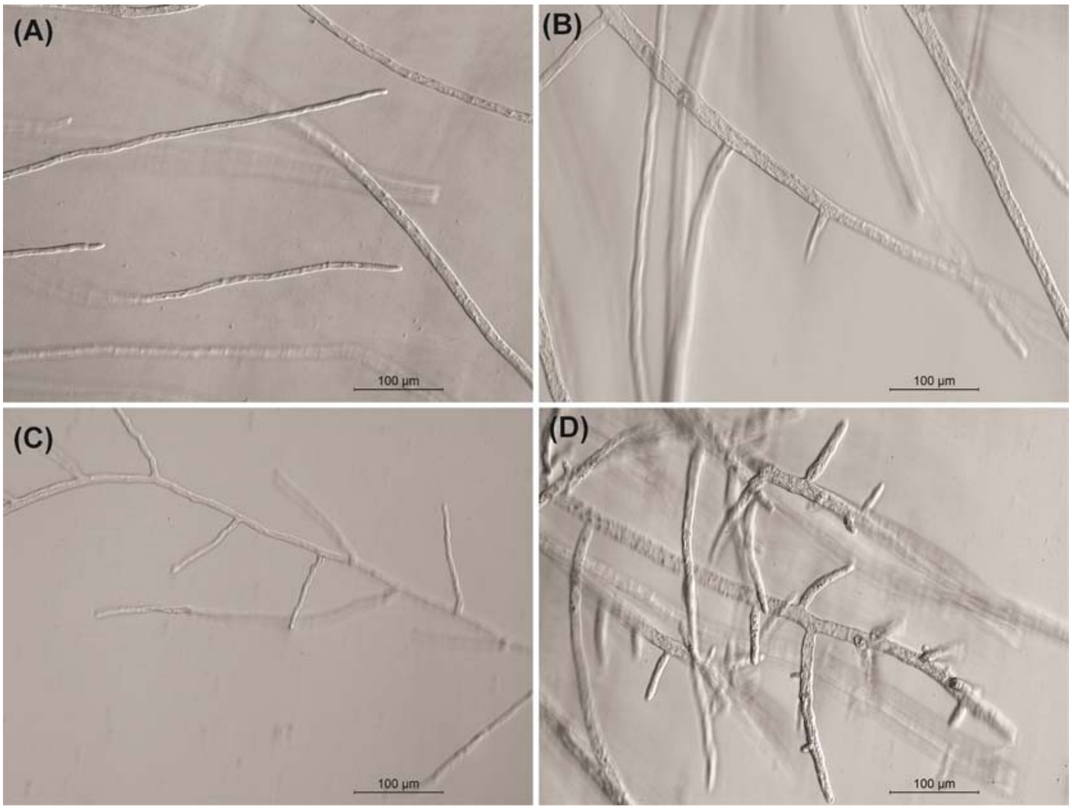

Supplementary Figure S3: Effect of different concentration of Triclosan on the level of hyperbranching in *S. parasitica* hyphae after 24 h culture at 24°C in Machlis medium. A: No Drug, B: Triclosan (1 µg/ml), C: Triclosan (2 µg/ml), D: Triclosan (4 µg/ml). Scale bars =100µm.
